# Her Heart's Mistake

**Published:** 1889-07-27

**Authors:** E. D. Cumming

**Affiliations:** Author of "An Ordeal by Silence"


### Her Heart's Mistake.

By E. D. Cumming, Author of "An Ordeal by Silence."

*(Continued from page 253.)*

### Chapter XV.—Kate does "Something Useful."

The tennis court and the club were for the present untrodden ground to Miss Harrison, and as the temporary absence of some of the medical staff laid a heavier burden of work on her father than usual, she rarely saw him until he came home to dinner, tired out with his labours at the hospital. She was therefore left much to her own devices, for her friends could not always be at her side; and the craving to find some useful occupation for her active mind grew stronger daily. She conceived a happy thought at last, and it promised to fulfil her requirements so satisfactorily that she wondered the idea had not occurred to her before. Dr. Harrison, like many other residents in the station, made a regular practice of sending the papers he received by every weekly mail from England to the patients under his care. Why should she not go up to the hospital every day for a few hours and read to the men? There could be no possible objection to her presence in the fever and accident wards, and she could thus employ many of the long hours now so sadly wasted over work she felt to be trivial and useless. She did not lose time in setting about the matter, and spoke to her father on it the same day.

"You are undertaking a much harder task than you think," was the Doctor's reply; "but if you would like to do it, those poor fellows will appreciate your visits more than anything we can do for them. There are no cases in the infectious wards now, and of course you wouldn't be allowed to go near them if there were; but you can go to any of the others. Don't overdo it; you will find that when you take up your post by one bed, every man in the ward will be straining his ears to listen to you, and you will be tempted to overtax your voice."

"What books should I begin with?" enquired Kate.

"I suspect you will find that the men's tastes vary considerably; you will probably find that highly-spiced sensational literature will give most satisfaction. Don't leave any books up there that you set value on, as the British soldier is not a careful reader, and punishes backs and covers pretty severely."

Kate spent that evening selecting twenty or thirty volumes of widely differing contents to form her library. Her aunt had been an omnivorous reader, and she found a plentiful stock of novels, good, bad, and indifferent, on the shelves in her dead relative's room. She was not guided in her choice by her own tastes; her purpose in taking up this employment was to give pleasure, and she was prepared to wade through anything which would serve that end, irrespective of her likes and dislikes.

The following afternoon saw her on Baroness on her way to the hospital, followed by her syce, carrying a load of books in his cloth. She set out to begin her first day's work with some little trepidation. She pictured herself standing on the threshold of a long ward with a row of faces turned eagerly towards her from the pillows on either side, and felt that it would be embarrassing to have to choose one patient, and so disappoint all the others. She was received in the hall, however, by Dr. Barrow, who took upon himself to suggest that she should begin in the "accidents," and she followed him upstairs, considerably relieved at having found someone to smooth over the difficulty which had haunted her during the ride up from the corner bungalow.

The ward into which Dr. Barrow led her was a large airy room, with bare floor and whitewashed walls, so dreary and prison-like that Kate's heart sank within her. At the far end, near the windows, the punkahs were waving lazily over four beds, which were the only ones occupied out of the eighteen or twenty in the apartment.

"There are four men close together here, you see, Miss Harrison," said her guide, laying down the books he had carried upstairs for her; "so you will be able to read to them all at once. I'll send up an orderly to move that bed, so that you can have your chair under the punkah."

Kate was not greatly prepossessed with the looks of the invalids; they all stared at her in mute curiosity, until Dr. Barrow addressed them on her behalf.

"This lady has come to read to you for an hour," he said; "would you like that?"

"Yes, sir, we'd like it fine," answered the man in the nearest bed, shyly; and the others echoed the sentiment more or less promptly. Hospital life was a pleasant enough change for a few days, but weeks spent in bed, sleeping, eating, and talking, palled upon the laziest, and though they responded somewhat sleepily, Kate saw that she was a welcome visitor.

"You must treat them just like children," said the Doctor in a low tone, as two attendants moved a cot to one side and placed a chair under the low hung punkah. "Talk to them as you would to intelligent little boys."

Kate hastily racked her brain to think which one of her assortment of books would meet with most acceptance from the men, who were all young, under twenty-four or five years of age at most. She sat down and turned over the little heap on the floor in some perplexity.

"What *am* I to give them?" she wondered. "'Far from the Madding Crowd'? I'm afraid that wouldn't be quite their style. Something of Ballantyne's would be the thing. Ah, that will do;" and she picked out a broken-backed copy of "Life on the Line," and cleared her throat to begin, while the men craned forward to listen. She opened the volume and selected a chapter at random, rightly thinking that so long as she read something interesting it did not much matter whether she commenced at one end of the book or the other. She hit upon that exciting account of the lunatic's elopement with an engine, and the pursuit; and was soon gratified to notice that her hearers were listening in rapt and open-mouthed attention.

"That's a A-1 book," said the spokesman approvingly, when she finished the chapter; and his comrades murmured a cordial assent. "You'll read a little more, likely?"

Kate read another chapter, and received a sincere but informal vote of thanks, coupled with a request to return soon, when she had done. Then, as the light was rapidly dying away, she put aside the book which had given such profound satisfaction, and proceeded to question the men upon the misadventures which had brought them to the hospital.

One had had his leg broken by a bullock-cart passing over it, and had been laid up for a month; another had been "knocked about" by a commissariat bullock, whose ferocity he described in language graphic and concise. The other two were incapacitated by minor injuries, but hoped that now Kate was coming to read to them they would not be sent back to duty for a long time to come. She went round to each bedside to shake hands before she went away, and took her departure with promises to come again next day. An offer to leave "Life on the Line" with them was accepted doubtfully, as seeming to portend that she did not mean to return.

"We likes to see you, ma'am," was the suggestive hint tendered by one gallant invalid, and the others repeated the words with convincing warmth. And Kate felt that she had found an occupation which was at once pleasant and pleasing.

She went up earlier the next day and spent an hour in the accident ward; but she was warming to her work, and was anxious to extend her favours to the other sufferers in the hospital; so she left, followed by four pairs of wistful eyes, and marched boldly in the first open door on the same landing.

She singled out a man who occupied a cot in one corner remote from the other inhabitants of the ward, and sat down beside him at once. He gratefully accepted her offer to "read a story" to him, and, as he could not suggest what kind of story he preferred, she was fain to attempt an appeal to his feelings through "The Chaplain of the Fleet." She lowered her voice to prevent the men in the distant beds striving to hear her, and read half of the first chapter wishout interruption; but as she paused to separate two leaves which had stuck together, her auditor called her to account.

"Yon's a funny book."

"Don't you like it?" enquired Kate.

"I'm not sayin' as I *don't* like it, but if so be's you've got a murder story, and would be willin'——."

"Very well," said Kate, "I'll read something else."

She selected a volume of Allan Edgar Poe's bloodcurdling tales, and treated him to that legend of the murdered wife's body being brought to light through the agency of the cat which the murderer had accidentally walled up with it. "I hope this is strong enough," she said to herself as she came to the climax. "If it isn't, I really don't know what will satisfy him."

It proved quite "strong enough" for her hearer's morbid taste, however, for he hung upon hei words with breathless interest until she reached the end and closed the book.

"Well, how do you like that story?" she asked, as she put it down.

"It beats the noospapers," said the invalid, in awestruck accents, "beats 'em holler. You don't see them things in noospapers."

It chanced one evening, when she was riding homewards in the rapidly-darkening twilight, that she saw Mr. Warren on the road in front of her, and pushed on to overtake him. He had given up his after-dinner visits to their house, and Kate had not seen him since the occasion upon which he had been so unusually silent. She had been forced to conclude that something she said had given him offence, and had often wondered what it could have been. However, here he was, so she drew up beside him, and with her natural frankness asked him the question point-blank.

"Good-evening, Mr. Warren," she began. "I am glad to have met you, as I have it on my conscience that I must have given you some cause of offence last time you came to see us. If I did, I hope you will tell me what it was."

It was too dark for her to see how his face changed colour, but he denied so formally and clumsily that she had offended him that she was more than ever convinced she had erred in some way or another.

"It is a long time since you have been in to see papa," she said, after a little awkward pause. "Why have you given up coming in to talk to him?"

All the uncouth diffidence and hesitation which had prejudiced her against him at the very beginning of their acquaintance seemed to take possession of him as he answered, "I've been very busy; I've had a great deal to do; so much work in the evenings now," he stammered.

It was the excuse he had given times without number in the days when he wanted to escape a dance or "at home" in London, but he had forgotten that it would not hold water in this country. Kate did not press him further, taking it for granted that he did not want to come; but she was anxious to re-establish the friendly understanding which used to exist between them, and began to tell him about the employment she had found in the hospital.

"I have discovered a new amusement, Mr. Warren," she said. "I go every afternoon to read in the wards, instead of strumming away at the piano or driving about the roads."

"I felt sure you would find some way of using your spare time," he said cordially, recovering his usual pleasant manner at once.

"Do you think it is useful?" she asked, wondering why her heart bounded at his implied praise.

"Most assuredly; I could not imagine a more kindly and beneficial occupation, or one which you could take up with greater advantage."

"I enjoy it immensely, and the men seem to like it too."

"I can well believe it," said Warren.

"It's so odd, the strange tastes they have," pursued Kate. "I was reading Russell's 'Letters from the War' to one man this afternoon, and the next would have nothing but 'Ralph the Bailiff.' They know me so well now that they ask for their favourite books without hesitation."

"How do you find out their proclivities, Miss Harrison? I should think uneducated men found a difficulty in making a choice."

"O, some of them are very easy to please. Only the other day I came to a new man, and asked him what he would like. 'Somethin' about lords and ladies, if you please, ma'am,' he said at once. So I had to take a severe dose of 'Strathmore,' which charmed him. It's not very elevating, but my aim is only to amuse."

"I don't suppose that any one man spends a long enough time in the hospital to enable you to try and mould his tastes to any extent?"

"There's one very intelligent man there now, a corporal, with a broken arm. I think I shall try and wean him from his beloved murder stories to something more refined and improving. How do you think I should set about it?"

"Its very difficult to suggest a method. Do his sympathies seem to lie with the criminal hero always?"

"No, I think not; but he gets quite excited over the most *awful* trash sometimes."

"It's just the craving for mental excitement we all possess in some shape or another. I fancy if you tried him with a book of thrilling adventure, you would find it give equal satisfaction."

"I had not thought of that; I'll give him a little of a book on Arctic exploration next time."

"Do you find that the general inclination is towards the sensational, Miss Harrison?"

"No, I can't say I do. One or two of the men like history better than anything else, and they do what the others don't seem to care about doing—that is, read it for themselves."

"The others prefer that you should read to them?"

"Yes; I daresay long words puzzle them."

Warren thought that it showed their good taste, but did not say so.

"It is curious what some men select to read for amusement," he said, after a pause. "I knew a business man in the City once, a remarkably clever man, too, who preferred a 'sixpenny novelette' to the best fiction published.

"How do you account for it?" enquired Kate.

"I suppose that being busy all day, he found the lightest possible literature the most palatable to a tired brain."

"And yet the idlest people read the greatest twaddle as a rule."

"I don't know that; I have noticed that men in this country read more solid books than at home. They have more time on their hands, and appreciate matter that demands closer attention than a novel, for the sake of the mental exercise."

They had reached the corner where Kate must leave him by this time; but before she bade him good-night something impelled her to try and get him to recommence his visits to her father.

"Mr. Warren, I hope that you are not going to give up coming in to sit with papa. I should be more vexed than I can tell you if I thought I had said anything to deter you when it gives us both so much pleasure."

"I assure you that you have said nothing, Miss Harrison," he replied, in a strangely low voice.

"Then let us see you again soon," she said, kindly.

They separated, and Kate rode into the compound with an inexplicable sense of relief at having "made it up" with him, and began to speculate how long it would be before he came again.

*(To be continued.)*

